# Impact of Clinical Pilates Exercise on Pain, Cardiorespiratory Fitness, Functional Ability, and Quality of Life in Children with Polyarticular Juvenile Idiopathic Arthritis

**DOI:** 10.3390/ijerph19137793

**Published:** 2022-06-25

**Authors:** Alshimaa R. Azab, FatmaAlzahraa H. Kamel, Maged A. Basha, Saud M. Alrawaili, Ghfren S. Aloraini, Sahar M. Hassan, Najlaa F. Ewais, Ragab K. Elnaggar

**Affiliations:** 1 Department of Physical Therapy and Health Rehabilitation, College of Applied Medical Sciences, Prince Sattam bin Abdulaziz University, Al-Kharj 11942, Saudi Arabia; s.alrawaili@psau.edu.sa (S.M.A.); rke_pt2001@yahoo.com (R.K.E.); 2 Department of Physical Therapy for Pediatrics, Faculty of Physical Therapy, Cairo University, Giza 12613, Egypt; 3 Department of Physical Therapy for Surgery, Faculty of Physical Therapy, Cairo University, Giza 12613, Egypt; dralzahraafatma@cu.edu.eg; 4 Department of Physical Therapy, College of Medical Rehabilitation, Qassim University, Buraidah 51452, Saudi Arabia; bashamaged@gmail.com (M.A.B.); smmh007@yahoo.com (S.M.H.); 5 Department of Physical Therapy, ElSahel Teaching Hospital, General Organization for Teaching Hospitals and Institutes, Cairo 11697, Egypt; 6 Department of Clinical Laboratory Sciences, College of Applied Medical Sciences, Prince Sattam University, Al-Kharj 11942, Saudi Arabia; g.aloraini@psau.edu.sa; 7 Department of Physical Therapy, Cairo University Hospitals, Cairo University, Cairo 4240310, Egypt; 8 Department of Basic Sciences, Faculty of Physical Therapy, Cairo University, Giza 12613, Egypt; najlaa.ewais@pt.cu.edu.eg

**Keywords:** juvenile chronic arthritis, physical rehabilitation, exercise, Pilates-based core strengthening, cardiopulmonary fitness, physical performance, health-related quality of life

## Abstract

Objective: This study intended to examine the effects of Pilates exercise on pain, cardiorespiratory fitness, functional ability, and quality of life in children with polyarticular juvenile idiopathic arthritis. Methods: Forty children with polyarticular JIA aged 10–14 years old were randomly allocated into two groups: the control group (*n* = 20) received conventional physical therapy (CPT), and the experimental group (*n* = 20) received clinical Pilates exercises combined with CPT. Patients in both groups received their program three times/week for 3 months. Pain, cardiorespiratory fitness, functional ability, and quality of life were assessed through the visual analogue scale, cardiopulmonary exercise test, 6 min walk test, and PedsQL scale, respectively, just before and after treatment. Results: Pain (*p* = 0.001), cardiorespiratory markers (all *p* < 0.05), functional ability (*p* = 0.002), and overall quality of life (*p* = 0.007) improved significantly in the experimental groups compared to the control group. Conclusion: Incorporating Pilates exercises into CPT is likely more effective for decreasing pain intensity, improving cardiorespiratory fitness, augmenting functional ability, and promoting quality of life in children with JIA than CPT alone.

## 1. Introduction

Juvenile idiopathic arthritis (JIA) is one of the most common chronic childhood inflammatory diseases and is characterized by permanent joint inflammation attributable to immune system disturbance [1]. The inflammation starts before the patient reaches the age of 16, and the symptoms remain for at least 6 weeks [2]. JIA affects about 1 in every 1000 children of both sexes, with a greater incidence among girls. JIA comprises a heterogeneous group of disorders (specifically, oligoarticular, polyarticular, and systemic JIA), all of which show signs of joint inflammation, but their clinical presentation, illness progression, and prognosis are different [3]. Children with polyarticular JIA (i.e., have arthritis in more than five joints) have an extended refractory course of active disease and a greater risk of joint damage in comparison to children with other JIA subtypes [4].

Children with JIA often present with pain, morning stiffness, and swelling in the involved joints. They also experience muscle weakness and atrophy around the inflamed joints and activity limitations [5]. Moreover, patients with JIA have been shown to have a low aerobic fitness level, which results from a combination of factors related to the disease pathology and the usage of steroids [6]. Other possible causative factors of diminished aerobic capacity are reduced physical activity levels and physical deconditioning arising from pain, muscular weakness, joint stiffness, and decreased cardiac output [7].

Impaired aerobic fitness and lower levels of physical function during the active stage of the disease lead to functional declines among children with JIA [8]. Further, even during the inactive phase, fear of exercises and the belief that they are detrimental to the joints as well as a lack of understanding about their benefits results in functional limitations. Furthermore, social isolation, which is frequent in chronic diseases, may contribute to inactivity and decreased functional ability in JIA patients [9]. The chronicity of the disease, physical activity difficulties, and pain are all known to reduce health-related quality of life (HRQOL) among children with JIA and their caregivers [10]. According to Haverman et al. (2012), children with JIA showed a decline across the core quality of life domains—the physical, psychological, and social domains [11]. 

Therapeutic exercises play an essential role as a non-pharmacologic treatment for children with JIA. They have been effectively used to reduce pain, improve muscular function, increase physical activity, and promote quality of life in children with chronic musculoskeletal disorders [12]. Pilates is a type of exercise that focuses on developing stability, muscle control, muscle strength, flexibility, respiration, and posture by combining body–mind–spirit interaction [13]. Clinical Pilates exercise is usually perceived as an enjoyable activity by children with JIA; thereby, it can help them be more active for extended periods without causing discomfort while also allowing them to control their breathing and avoid becoming fatigued [14].

Although evidence is mounting for the beneficial effects of Pilates exercises on the cardiorespiratory fitness and physical activity level in healthy people and different patients (such as COPD, obesity, ankylosing spondylitis, and cystic fibrosis patients) [15,16,17], studies that have examined its effects in children with JIA are very limited. As such, the purpose of this study was to compare the effect of Pilates exercises plus conventional physical therapy versus conventional physical therapy alone on pain, cardiorespiratory fitness, functional status, and quality of life in children with polyarticular JIA. We hypothesized that the combined intervention would result in more favorable changes in all outcome measures compared to conventional physical therapy alone.

## 2. Materials and Methods

### 2.1. Study Design

This randomized, controlled clinical trial was conducted between August 2020 and July 2021 in the physical therapy outpatient clinic at the College of Applied Medical Sciences, Prince Sattam bin Abdulaziz University (PSAU), Saudi Arabia. PSAU’s Physical Therapy Research Ethics Committee (No: RHPT/0020/0035) provided ethical approval. Study measures were performed in agreement with the Declaration of Helsinki 1975 ethical standards for medical research. Before enrollment, all procedures were fully demonstrated to all patients and their caregivers. They were then requested to sign a consent form. A researcher who was not aware of treatment assignment evaluated pain severity, cardiorespiratory fitness, physical activity level, and quality of life before and after the intervention. This trial was registered at ClinicalTrials.gov (ID: NCT05231057).

### 2.2. Subjects

Patients were enrolled from the Maternity and Children’s Hospital and King Khalid Hospital (Pediatric Rheumatology Department) in Al-Kharj, Saudi Arabia. Inclusion criteria were as follows: polyarticular JIA according to the classification of International League of Associations for Rheumatology [18], age range from 10 to14 years old, and stable medical treatment. Subjects were excluded if they had contractures or congenital anomalies, history of surgery or systemic disease, or cardiorespiratory co-morbidities and participated in regular exercises or sports activity.

### 2.3. Assignment Procedure

Forty patients met the criteria. Following the baseline examination, they were divided into two equal-sized groups at random. Conventional physical therapy (CPT) was administered to the control group (*n* = 20), while Pilates exercises plus the conventional physical therapy program were administered to the experimental group (*n* = 20). An independent researcher used a simple randomization approach in which sealed non-transparent envelopes included a code for one of the two experimental sets (control or experimental). These were drawn to determine intervention assignment for each participant. 

### 2.4. Outcome Measures

Pain, cardiorespiratory fitness, functional ability, and quality of life were evaluated just before and directly after the treatment by a pediatric physical therapy researcher who was blinded to the treatment procedures of both groups.

### 2.5. Primary Outcome Measures

#### 2.5.1. Pain Assessment

Pain severity was measured using a 10 cm visual analog scale (VAS), with 0 demonstrating no pain or discomfort and 10 indicating the strongest pain experience. Participants were instructed to cut the line at a point that represented how much pain they felt at rest and/or during activity [19].

#### 2.5.2. Cardiorespiratory Fitness Assessment

A graded cardiopulmonary exercise test was used to assess cardiopulmonary fitness. An electromagnetic cycle ergometer with electronic braking (ER900; Ergoline, Bitz, Germany) was used to assess the peak oxygen uptake (peak VO_2_), maximum heart rate (HRmax), and breath-by-breath minute ventilation (VE) according to the McMaster incremental cycle protocol [20]. Before starting the test, a simple demonstration was provided to each patient to familiarize them with the test measures. They were advised to avoid heavy meals or drinking caffeine three hours before testing and to wear light, comfortable clothing and shoes during test procedures. The test consisted of a conventional cycling rate of 50–70 rpm, beginning with a 25-watt load and increasing by 25 or 50 watts at 120 s breaks according to the tallness and sex of the participant. A 3 min active rest period at a training intensity of 25 watts was allowed at the end of the test, followed by a passive rest period of 3 min.

### 2.6. Secondary Outcome Measures

#### 2.6.1. Functional Ability Assessment

Functional ability status was assessed using the Childhood Health Assessment Questionnaire (CHAQ-38). It assesses nine domains: dressing, grooming, getting out of bed, eating, ambulation, self-hygiene, grip reach, activities, and extra-curricular events. The results were transformed to a CHAQ disability score ranging from 0 to 3 (a higher mark indicates a higher level of disability) [21].

#### 2.6.2. Quality of Life

The self-reported Pediatric Quality of Life Inventory was used to assess quality of life (PedsQL). PedsQL is a 23-item multidimensional measure of health-related quality of life (HRQL) in children and adolescents and is divided into 4 areas (physical: 8 items, emotional: 5 items, social: 5 items, and school functions: 3 items). This questionnaire has been approved for usage with kids and adolescents aged 2 to 18 years old [22]. On a 5-point scale, each item was rated (0 means never, and 4 means almost always). Items were converted to a 0–100 scale on a linear scale (0 = 100, 1 = 75, 2 = 50, 3 = 25, and 4 = 0). In this study, the physical health summary score was calculated by summing physical function item scores and dividing the total score by the number of rated items. Psychosocial health summary score was calculated by summing item scores of emotional, social, and school functions then dividing the total score by the number of rated items. The total summary score was calculated by summing all item scores in all domains (physical and psychosocial) and dividing the total score by the number of rated items). Greater scores equated to a higher quality of life.

#### 2.6.3. Conventional Physical Therapy Program (CPT)

All children in the two groups underwent the conventional PT program (40 min per session, 3 sessions each week over 3 months), which was delivered by a pediatric physical therapist. The key priorities were reliving pain, increasing flexibility and mobility, improving muscle strength, and promoting proprioceptive awareness as well as increasing aerobic fitness. In general, the program consists of pain reliving modalities, stretching exercises, active range of motion activities, weight bearing activities, aerobic exercises on a treadmill or a bicycle, and isometric exercises [23,24,25].

#### 2.6.4. Pilates Exercises

Pilates exercises (25 min each session, 3 sessions each week over 3 months) were administered to patients in the experimental group by a pediatric physical therapist. Mats, Pilates bands or elastic bands, and Pilates ball are some of the several types of equipment used in Pilates exercises to attain various goals [26,27]. Detailed description of Pilates exercises is shown in Table 1.

#### 2.6.5. Sample Size Calculation

The analysis was conducted using G*Power program version 3.0.10 (Neu-Isenburg, Germany). A group sample size of 16 (totaling 32 for both groups) was needed to achieve 81.23% power to reject the null hypothesis of zero effect size on pain intensity when the population effect size is too small (Cohen’s *d* = 1.04; as calculated in a pilot study of eight children who received similar interventions). The significance level was 0.05 using a two-sided independent *t*-test. To account for a potential dropout rate of 20%, the sample size was increased to 40 (that is, 20 in each group).

#### 2.6.6. Data Analysis

Data analyses were conducted with SPSS version 21 (SPSS Inc., Chicago, IL, USA). The Kolmogorov–Smirnov test was used to determine data normality. An independent *t*-test (for continuous data) and a Chi-square test (for categorical data) were used to assess baseline differences between the IMT and control groups. The analysis of covariance (ANCOVA) test was used to calculate the post-treatment differences between the two groups (for primary and secondary outcome variables). To compare the two groups, the pre-treatment values of the dependent outcome variables were employed as covariates. The partial eta-squared equation was used to calculate the effect size (ES) for the significant ANCOVA. For all statistical tests, the level of significance was set at *p* < 0.05.

## 3. Results

### Recruitment and Retention of Participants

**Figure 1:**
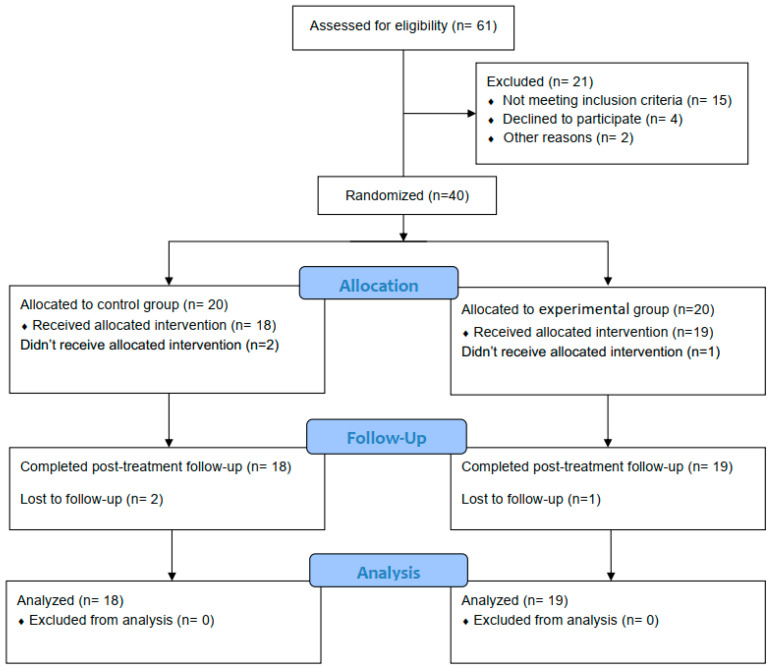
Participants’ CONSORT flowchart.

The children involved in the current study were screened for eligibility. Out of 61 possibly eligible children, 40 participants met the criteria for inclusion and were randomly assigned to either the control or the experimental group. Three children did not complete the study (one from the experimental group and two from the control group). The CONSORT flow chart is explained in Figure 1. The baseline demographic and clinical characteristics for each group are presented in Table 2. Additionally, the primary/secondary outcome measures in both groups at the pre-treatment point are shown in Table 3.

Table 4 shows the post-treatment differences in the outcome variables between the experimental and control groups, which were controlled for pre-treatment values. In terms of pain intensity, there was a large significant difference between both groups in regard to the VAS scores (F (1, 34) = 12.23, *p* = 0.001, ES = 0.92), which were in favor of the experimental group. In addition, there were large significant differences in the cardiopulmonary fitness between the two groups, as measured by the peak VO_2_ (F (1, 34) = 8.45, *p* = 0.008, ES = 0.81), VE (F (1, 34) = 11.16, *p* = 0.002, ES = 0.90), and HRmax (F (1, 34) = 6.39, *p* = 0.004, ES = 0.84), where the experimental group showed better cardiopulmonary fitness than the control group.

Further, there was a large significant difference between the two groups concerning their functional status (F (1, 34) = 10.75, *p* = 0.002, ES = 0.89), as the mean CHAQ score was lower in the experimental group in comparison to the control group. Still, there were significant differences in the quality-of-life domains between both groups: the physical dimension (F (1, 34) = 7.21, *p* = 0.011, ES = 0.74), psychosocial dimension (F (1, 34) = 5.17, *p* = 0.029, ES = 0.59), and total scores (F (1, 34) = 8.20, *p* = 0.007, ES = 0.79) were all in favor of the experimental group.

## 4. Discussion

The current study was designed to evaluate the influence of Pilates exercises besides conventional physical therapy on pain, cardiorespiratory fitness, physical activity level, and quality of life in children with polyarticular JIA. Our results revealed that combining Pilates exercises with a conventional physical therapy program yielded favorable outcomes regarding the reduction in pain perception, the improvement of cardiorespiratory fitness, increasing the physical activity level, and the enhancement of quality of life.

The role of Pilates exercises in relieving pain may be attributed to the breathing exercises, which are a main component of Pilates, as breathing exercises improve lung capacity, blood oxygenation, and circulation. Pilates also encourages the brain to release normal endorphins and opiates, which reduces pain and stress [28]. Increased body flexibility may be a causative factor of decreasing pain as reported by Segal et al. [29], who reported that Pilates exercises increase flexibility, reduce muscle tension during exercise, reduce muscle aches, improve physical performance, and reduce the energy cost of joint movements. Our results are consistent with review research by Wells et al. [13], which concluded that Pilates exercises decrease pain intensity and increase functional ability in patients with low back pain more than traditional physical therapy programs. The results of this study are in agreement with research conducted by Unal et al. to examine the consequence of clinical Pilates exercises on JIA children, concluding that there was a positive impact of Pilates exercises on decreasing pain intensity and improving function and overall health status [14].

One possible explanation for the constructive effect of our results is the weekly exposure to the exercises, as the two interventions (Pilates + CPT) were delivered to improve cardiorespiratory fitness. The strengthening of the lumbopelvic region and core muscles that occurred during the exercises may have led to a better movement pattern in the limbs and better strength in the expiratory muscles [30]. Another cause may be due to the increased mobility of the rib cage and intercostal muscles during breathing exercises, which can lead to a more efficient breathing pattern, improved ventilation efficiency, greater flow of oxygenated blood into muscle, and improved pulmonary functions [31].

The results of our study are also in line with the study conducted by Souza et al. to investigate the effect of cardiorespiratory adaptation to Pilates training. They concluded that 15 successive weeks of Pilates training has a positive impact on cardiorespiratory factors such as VO_2_ max and ventilatory thresholds in sedentary women with decreased respiratory capacity levels [32]. Our results are also in agreement with Niehues, who concluded that Pilates exercises increase the strength of the abdominal muscles, which could be the cause of improvements in diaphragmatic function, enhancements in respiratory function, and improvements in cardiorespiratory fitness [33].

Our results also showed that 3 months of clinical Pilates exercises in adolescents with JIA improved their functional status. This improvement may be associated with reduced pain intensity and improved aerobic capacity. We verified this claim through a finding of a study by Limenis et al., who studied the association between physical activity levels and functional status with pain in JIA children and concluded that there was an inverse relationship between pain severity and functional activity level [34]. A study by Lelieveld et al. studied the functional activity in adolescents with JIA, and the results of this study show that a higher level of functional activity is related to a low pain level and a high level of well-being. PA is also associated with fitness level, as a decreased fitness level might be the cause for deconditioning and lower function [6].

The results of our current study revealed that there was enhancement in the HRQOL in children with JIA, and these results are consistent with the results of Calik et al., who stated that clinical Pilates exercises increase mobility in children and adolescents with JIA, making them more active in daily activities. This has enables them to improve their overall quality of life [27]. Our results are also in agreement with Mendonça et al., who concluded that the physical and psychosocial aspects of the HRQOL were positively improved in groups treated with Pilates exercises when compared to groups treated with a conventional physical therapy program [35].

Although the results of this study are mostly positive, there are some limitations that affect the generalizability of the results. First, the lack of long-term follow-up means further research is required to study the effects of Pilates exercises after 6–12 months. Second, a small sample size and relatively low power mean further studies are required to examine the influence of Pilates exercises on a large sample, thus resulting in more solid conclusions. Special consideration should be given when generalizing these findings, as this study was conducted on patients with only one specific type of polyarticular JIA and in one specific age group. As such, more research is desirable to explore the impact of Pilates exercises combined with a conventional physical therapy program on other types of JIA and on different age groups of children with JIA.

## 5. Conclusions

Notwithstanding the relatively limited sample, this work suggests that the incorporation of Pilates exercises into physical therapy programs is likely an effective strategy for decreasing pain intensity, improving cardiorespiratory fitness, augmenting the physical activity level, and promoting HRQOL in children with JIA. However, large randomized controlled trials could provide more definitive evidence.

## Figures and Tables

**Table 1:** Pilates exercises.

Exercises	Description
**Hundred exercise**	Child is in supine position with hips and knees at a right angle. They raise their head and shoulders up and extend both upper limbs. They breathe in and out deeply, contract abdominal muscles, and move their upper limbs up and down 100 times. Repeat 5 times for 3 sets.
**One leg stretch**	Child is in supine position with hips and knees in right angle with head and shoulders up from the ground. They breathe in and out deeply while bringing their left knee to their chest and stretching their right lower limb. They then alternate legs. Repeat 5 times for 3 sets.
**Hip twist**	Child sits on mat with both legs straight forward, and both hands behind their back. The child breathes in and out deeply, moves both legs up, and circles the legs toward the right and the left. Repeat 5 times for 3 sets.
**Knee stretches**	Child kneels on carriage with both feet touching shoulder breaks and grasping foot-bar with hands. They inhale and exhale deeply, stabilize their back and arms, and then extend hips and knees to move the carriage out as far as possible while maintaining a straight pelvis and spine. Repeat 5 times for 3 sets.
**Arm openings**	Child is in a side-lying (right side) position with hips and knees flexed to 90° and palms facing each other as the right upper arm rests on the mat and the left arm on top of it. They breathe in and out deeply and raise their left arm up toward the ceiling and rotate their head and neck to follow the left upper arm and return the arm back to the starting position. Repeat on the opposite side. Each side repeated 5 times for 3 sets.
**Plank**	Child is in prone position with head, trunk, and lower limbs straight and upper arms parallel to their elbows. Shoulders are wide apart, both knees are in full extension, and toes are pointed in planter flexion. They breathe in and out deeply and hold this position for 30 s.
**Standing footwork**	Child is in a standing position with heels together and arms crossed in front of chest. They raise up on their toes as far as possible and then lower their body while keeping the back in neutral position. They then their bend hips and knees while keeping their heels together and opening both knees. Repeat 5 times for 3 sets.
**Squat**	Child is in a standing position on a Pilates band and grasping both ends of the band with their hands with straight elbows and breathing in and out deeply as they squat to a 90° flexion in the knees and elbows and then stand up while pulling the band. Repeat 5 times for 3 sets.
**Swimming with stabilization ball**	Child is in a prone position and holds a Pilates ball with out-stretched hands with lower limbs slightly elevated and abducted. They breathe in while moving their upper limbs upward with the ball and move their lower limbs up and down reciprocally. They breathe out and lower their upper limbs while their lower limbs move up and down. Repeat 5 times for 3 sets.
**Wall Squat Rolls**	Child is in standing position against the wall with a Pilates ball in the middle of their back with feet facing away from the wall. They breathe in deeply and squat down slowly on the ball and then breathe out and move slowly back up. This counts as one repetition. Repeat 5 times for 3 sets.

**Table 2:** Demographic and clinical characteristics of children in the IMT and control groups.

	Experimental Group (*n* = 19)	Control Group (*n* = 18)
Age, years	12.32 ± 1.67	11.56 ± 1.46
Gender (M/F), *n* (*%*)	7 (36.8%)/12 (63.2%)	4 (22.2%)/14 (77.8%)
Weight, kg	36.47 ± 4.59	35.56 ± 4.20
Height, m	1.43 ± 0.06	1.33 ± 0.05
BMI, kg/m^2^	20.19 ± 1.72	19.98 ± 1.46
Age of onset, years	4.68 ± 0.58	4.56 ± 0.92
JIA duration, years	7.74 ± 1.48	7.17 ± 1.42
Joints involved, *n*	8.11 ± 1.24	7.55 ± 1.15
Rheumatic factor (+/−), *n* (*%*)	4 (21.1%)/(15, 78.9)	2 (11.1%)/16 (88.9%)
Corticosteroids, mg qod	172.89 ± 68.99	149.72 ± 56.10
Methotrexate, mg/week	16.74 ± 4.24	15.10 ± 3.67
Steroid injection, *n*	14.82 ± 7.80	13.28 ± 4.14

Data expressed as mean ± SD if continuous or as frequency (%) if categorical, M/F: male/female; BMI: body mass index; JIA: juvenile idiopathic arthritis.

**Table 3:** Primary/secondary outcome measures in the experimental and control group at the pre-treatment point.

	Experimental Group (*n* = 19)	Control Group (*n* = 18)
Pain intensity		
VAS	6.74 ± 1.15	7.17 ± 0.78
Cardiopulmonary fitness		
Peak VO_2_, mL/Kg/min	26.94 ± 1.59	25.99 ± 2.06
VE	73.15 ± 4.10	71.82 ± 5.31
HRmax, beat/min	182 ± 7	184 ± 5
Functional status		
CHAQ	1.40 ± 0.59	1.28 ± 0.50
Quality of life		
Physical	71.95 ± 5.94	70.14 ± 5.41
Psychosocial	72.54 ± 5.43	71.23 ± 6.10
Total score	72.25 ± 4.45	70.69 ± 3.99

VAS: visual analogue scale; peak VO_2_: peak oxygen consumption; VE: minute ventilation; HRmax: maximum heart rate; CHAQ: Childhood Health Assessment Questionnaire.

**Table 4:** Post-treatment differences in primary/secondary outcome measures between the experimental and control group: controlled for pre-treatment values.

	Experimental Group (*n* = 19)	Control Group (*n* = 18)	Sig.	Effect Size
Pain intensity				
VAS	4.57 ± 1.17	5.94 ± 1.11	0.001 *	0.92
Cardiopulmonary fitness				
Peak VO_2_, mL/kg/min	30.10 ± 2.56	27.90 ± 1.62	0.008 *	0.81
VE, L/min	78.31 ± 3.53	73.47 ± 7.56	0.002 *	0.90
HRmax, beat/min	196 ± 4	188.83 ± 10	0.004 *	0.84
Functional status				
CHAQ	0.78 ± 0.39	1.12 ± 0.40	0.002 *	0.89
Quality of life				
Physical	81.86 ± 4.24	75.76 ± 7.99	0.011 *	0.74
Psychosocial	79.34 ± 5.93	74.55 ± 6.28	0.029 *	0.59
Total score	80.59 ± 3.96	75.16 ± 6.55	0.007 *	0.79

VAS: visual analogue scale; peak VO_2_: peak oxygen consumption; VE: minute ventilation; HRmax: maximum heart rate; CHAQ: Childhood Health Assessment Questionnaire; Sig: level of significance: * *p* < 0.05.

## Data Availability

The authors declare that all relevant data supporting the findings of the study are available within the manuscript.
